# From theory to practice: insights and hurdles in collecting social media data for social science research

**DOI:** 10.3389/fdata.2024.1379921

**Published:** 2024-05-30

**Authors:** Yan Chen, Kate Sherren, Kyung Young Lee, Lori McCay-Peet, Shan Xue, Michael Smit

**Affiliations:** ^1^School for Resource and Environmental Studies, Dalhousie University, Halifax, NS, Canada; ^2^School for Resource and Environmental Studies, Faculty of Management, Dalhousie University, Halifax, NS, Canada; ^3^Rowe School of Business, Faculty of Management, Dalhousie University, Halifax, NS, Canada; ^4^Nova Scotia Department of Cyber Security and Digital Solutions, Halifax, NS, Canada; ^5^Department of Systems Design Engineering, Faculty of Engineering, University of Waterloo, Waterloo, ON, Canada; ^6^School of Information Management, Faculty of Management, Dalhousie University, Halifax, NS, Canada

**Keywords:** social media, Instagram, data collection, data ethics, secondary data, application programming interfaces, landscape research, visual methods

## Abstract

Social media has profoundly changed our modes of self-expression, communication, and participation in public discourse, generating volumes of conversations and content that cover every aspect of our social lives. Social media platforms have thus become increasingly important as data sources to identify social trends and phenomena. In recent years, academics have steadily lost ground on access to social media data as technology companies have set more restrictions on Application Programming Interfaces (APIs) or entirely closed public APIs. This circumstance halts the work of many social scientists who have used such data to study issues of public good. We considered the viability of eight approaches for image-based social media data collection: data philanthropy organizations, data repositories, data donation, third-party data companies, homegrown tools, and various web scraping tools and scripts. This paper discusses the advantages and challenges of these approaches from literature and from the authors' experience. We conclude the paper by discussing mechanisms for improving social media data collection that will enable this future frontier of social science research.

## Introduction

Social media has profoundly changed our modes of self-expression, communication, receipt and dissemination of information, construction of social bonds, and participation in public discourse and events (Lazer et al., 2009; Acquisti et al., 2015). In the first decade of the flourishing of social media, the potential value of social media data also caught the attention of researchers. Over the subsequent years, it has been consistently demonstrated that this data assists in our understanding of society and human behavior (Chen et al., 2023; Sherren et al., 2023). Early studies on the use of social media in politics confirmed the meaningfulness and power of the data, followed by research in various areas like business, communication, health, environment, and sociology (Savage and Burrows, 2007; Edwards et al., 2013; Procter et al., 2013; Chen et al., 2018), leveraging platforms such as *Twitter, Flickr, Weibo, Panoramio, YouTube, Facebook*, and *Instagram* (Ghermandi and Sinclair, 2019; Chen et al., 2023; Gone et al., 2023).

The Cambridge Analytica Scandal in 2018—triggered when *The New York Times* reported the data of millions of *Facebook* users were fraudulently accessed by a consulting company (Confessore, 2018)—was a major turning point that led to the current “post-API” (application programming interface) age, with social media platforms restricting or paywalling access to public search APIs, fine-grained location data, and more (Freelon, 2018). The majority of the most widely used platforms have transitioned toward a stricter and more commercialized policy, closing access to such data for public good research. *Instagram* closed their less-restricted access; instead, they issued two types of API for business app use only (Meta for Developers, 2023). This has an impact on research areas that place greater value on image data, such as landscape studies. *Meta* recently launched a new Content Library API in November 2023, an access-controlled space to work on Facebook and Instagram data rather than downloading complete copies, yet it has not been widely used (Meta, 2023). *X*, previously known as *Twitter*, closed their academic research API in 2023 after Elon Musk's acquisition and created three new versions of paid-API access (X Developer Platform, 2023). By contrast, the most popular short video platform, *TikTok*, has launched an application-based research API. Currently, the application is only open to US- and Europe-based researchers, but it may become available to all researchers in the future (TikTok for Developers, 2023).

Researchers are in an increasingly weak position with respect to social media data access (Zuckerman, 2023). John and Nissenbaum (2019) wrote that “researchers are ultimately dependent on tech companies for data and have to find a way to collaborate while serving the public interest and avoiding bias” (p. 3). The increased restrictions in APIs leave social media researchers grappling with non-public, legally ambiguous, and ethically gray approaches to collecting data, or push them toward impermanent types of data that hamper the detection of trends (Weller and Kinder-Kurlanda, 2015; Kinder-Kurlanda and Weller, 2020). Business-oriented users of data continue unencumbered, while access to data for public good is curtailed (Bruns, 2019; John and Nissenbaum, 2019; Acker and Kreisberg, 2020). Poletti and Gray (2019) observed that “academic research is now competing with market research, and it is no longer the dominant party when it comes to providing interpretations of society” (p. 265).

Social media imagery data, along with its geo-tags, is recognized for its value across diverse social science fields (e.g., environment, sociology, politics, health, etc.), though its collection can be complex due to the necessity of retrieving additional image files (Chen et al., 2023). In this paper, we considered eight approaches to image-based social media data collection: data philanthropy organizations, data repositories, data donation, third-party data companies, homegrown tools, and various web scraping tools and scripts. To manage the scope, we considered the viability of each approach for an energy landscape study in rural Canada. The case leverages our engagement in longitudinal research which helped us to understand the challenges after 2018. While the case study might not interest a broad research community, the insights garnered from the data collection process are universal because: (1) the framework of the eight approaches is consistent for all social media data, and (2) various tools examined in this paper can extract diverse data types, such as texts and videos, from different social media platforms. From the analysis of the approaches, this paper offers three main contributions. First, it tests these approaches for their feasibility in gathering *Instagram* data by geographic locations, providing insights for social scientists who are interested in leveraging social media data for place-specific questions. Second, it details advantages and challenges from the literature and from the authors' experience. Third, it raises the idea of a forward-looking solution for a research API, building on nascent efforts undertaken by social media companies and regulatory frameworks.

## Methods

The energy landscape project we used to assess the viability of these data collection approaches necessitated gathering *Instagram* posts depicting images of outdoor landscape use around hydroelectric dams and reservoirs in rural Canada. We identified eight social media data collection approaches from literature and practice to assess for their effectiveness, benefits, and drawbacks for the project. These approaches include the following:

***Data philanthropy organizations***: Entities that distribute data without charge. Many of them are based on industry-academic partnerships.

***Data repositories***: Entities that host data contributed by scholars for broader re-use.

***Data donation***: A relatively new practice in which individual users can request their personal social media archives and donate them to data repositories or research projects.

***Third-party data companies***: Commercial tools developed by third-party companies to monitor, retrieve, and analyze social media data.

***Homegrown tools***: Software tools for extracting social media data that are rooted in academic soil (i.e., made for researchers by researchers) and tend to charge more affordable rates and provide data in researcher-friendly formats.

***Web scraping tools—commercial***: Commercial web scraping tools provide the service of automatically extracting content from social media posts for profit.

***Web scraping tools—non-commercial***: Non-commercial web-scraping tools are collaboratively built and shared as open-source software online.

***Web scraping scripts (single-purpose)***: Software scripts developed by researchers for a specific research project, perhaps using a library or template.

## Results

Many approaches proved unsuitable for collecting *Instagram* data in our landscape research for diverse reasons (Table 1). This section outlines each approach's strengths and weaknesses, as per literature and our experiments, and clarifies why most failed.

**Table 1:** Advantages and limitations of social media data collection approaches.

**Approach**	**Examples**	**Advantages**	**Limitations**	**Hurdles from authors' experiences**
Data philanthropy organization	Facebook Ad library Social Science One	• Full access to a more complete dataset • Low or no legal risk • No monetary cost to the researcher	• Limited social media platforms • Delivery delays • Veto right reserved by social media companies • Requires an application • Limited research topics • Deadline restriction	• Instagram does not have such organizations to provide data • Landscape research is not a prioritized topic
Data repositories	Inter-university Consortium for Political and Social Research	• No monetary cost to the researcher	• Legal and ethical concerns of sharing data • No existing data available • Data disconnected from original context • Requires sustainable funding for the repository	• No existing data for our study cases
Data donation	Breuer et al., 2020	• Data include a wide range of user activities • No or low legal and ethical risk	• Requires recruiting participants (time-consuming and ethical review) • Complicated process • Limited size of data and response bias	• Limited time and budget to collect large-sized data
Third-party data companies	HootSuite Sprout Social	• User-friendly • Low legal risk	• High cost • Ill-suited data formats for research purposes	• Difficult to find a provider (they are business-oriented) • Limited budget
Homegrown tools	Netlytic Communalytic	• Well-suited data formats for research purposes • User-friendly • Affordable price	• Heavily depend on platforms' APIs • Not always well-maintained or self-sustaining	• Unable to collect data by geographic index due to API limitations
Web Scraping tools (commercial)	ScrapeStorm Apify	• Affordable price • User-friendly	• Incomplete dataset (export limit) • Ill-suited data formats for research purposes • Ethical and legal risk	• The tool we attempted had a maximum data export limit
Web Scraping tools (non-commercial)	Instagram scraper	• No monetary cost	• Not user-friendly and requiring programing skills • Inflexible and unstable (can stop working when the media interface changes) • Ethical and legal risk • Time consuming	• The tool we chose became non-functional halfway through
Web Scraping scripts (single-purpose)	github.com/Titration/ Ins-Scraping	• Well-suited data formats for research purposes • Flexible • Low up-front cost	• Ethical and legal risk • Time consuming • Requires programing skills • Must be customized for each research project and platform. • Unstable	• It took us 5 months to collect around 80,000 posts

### Data philanthropy organizations

The advantages include that researchers can have full access to a more complete dataset than would be available through other means without any legal risk, because the organization helps to build industry-academic partnerships through which social media researchers can obtain data directly from the company.^1^ To qualify, the study topics must be narrowly related to specified areas, such as the effect of social media on democracy for *Social Science One*, which eliminates most environmental and landscape research like ours. The scrutiny on applications is strict, and the veto right reserved by social media companies casts a long shadow over research independence (Bruns, 2019). Such organizations often only receive application submissions by deadlines, also leaving the data collection work less flexible and incompatible with fast-changing environmental and social issues.

### Data repositories

Data repository is an alternative that can reduce the influence of social media companies (Borgman, 2019; Acker and Kreisberg, 2020). However, largely due to legal and ethical concerns about sharing social media data, many researchers are cautious. Also, the specifications required for data collection for one study may make the data useless to others. Few scholars use the entirety of social media data during a period; most are looking for subsets in specific locations or referencing specific keywords or hashtags. Our research project is an example: there were no previous studies we could find that collected and shared *Instagram* posts from our target case areas. Keeping and delivering huge social media data can be financially, ethically and technologically difficult (Borgman, 2019), especially when most social science users will use a small (but unpredictable) fragment of that data (Chen et al., 2023). In repositories, data can become disconnected from their context and dealing with data duplicates can be troublesome (Weller and Kinder-Kurlanda, 2015). As such, data repositories may not yet be a solution for most research projects.

### Data donation

Data donation can provide a wide range of user activities including private messages and ephemeral content (Van Driel et al., 2022). However, since the donation system has not been fully developed, it typically requires researchers to find, contact and potentially compensate people first and ask them to follow the donation steps (Breuer et al., 2020). This may not benefit all kinds of research, especially those requiring large-sized data or not focusing on specific actors in a system. In addition, for research using social media data as a substitute for conventional approaches like survey and interview, data donation follows a more complicated but less mature process. It also introduces response bias on top of biases inherent to social media data, and there is not a clear research ethics regime in place for encouraging donations to a repository.

### Third-party data companies

Although free of legal concerns, purchasing data from these third-party companies can be expensive and yet not provide data well-suited for research purposes. We inquired with two *Instagram* partner companies to collaborate on data collection for our research project on the topic of hydropower landscape. Neither responded to our emails or web submission forms. It is easy to understand why: first, both companies are large and likely prefer large customers who can bring sizable revenue; second, their business is focused on marketing and advertising analytics and their tools are less applicable for research purposes.

### Homegrown tools

Homegrown tools for data collection can provide data in researcher-friendly formats at a reasonable cost. For instance, *Netlytic* is free for small datasets and has been used by many social scientists to extract social media data, especially pre-2018. A related product *communalytic* is affordably priced and includes access to historical Reddit data and has limited abilities to import other social media data (e.g., comments on a specific *YouTube* video). However, any such tools are heavily dependent on social media APIs which grant them no higher levels of access than the public. We used *Netlytic* in our original cross-sectional landscape studies to collect *Instagram* posts by geographic coordinates. When *Instagram* stopped supporting the geo-location index in their API, *Netlytic* terminated the service for *Instagram* data.

### Web scraping tools—commercial

Commercial scraping tools are often more affordable than third-party companies but can still be a big investment if a large-sized dataset is required. In our review of widely used scraping tools for *Instagram* data, most provided services by subscription at prices ranging from $5 to hundreds of US dollars per month, and there were limitations in terms of exported data size and formats (e.g., *ScrapeStorm* and *Apify*). Downloading images, which are increasingly critical to social media research (Chen et al., 2023) and to landscape studies, would result in additional charges. There is another concern on the completeness of datasets because most of these tools have a cap on data export amounts.

### Web scraping tools—non-commercial

Another type of scraping tool is the non-commercial ones, such as *Instagram Scraper* (GitHub, 2022). This tool is not as user-friendly as the commercial tools that operate on a graphical interface. Instead, it is code-based which requires users to have basic knowledge of and experience with Python to operate it. *Instagram Scraper* operated properly when we started to collect data in September 2020; however, it became quite unstable from February 2021 and there was no update of the tool until 1 year later. Open-source software is community-supported, which means a developer needs to be willing to contribute their time to ensure the software stays up-to-date with rapidly changing social media platforms.

### Web scraping scripts (single-purpose)

For researchers with (or with access to) sufficient technical expertise, self-developed scraping scripts can provide more flexibility and collect data that is well-suited to the scholarly research analysis they have planned. In our case, a local software developer agreed to help develop custom scraping scripts based on two Python packages –*Selenium* and *Instascrape* (our *Instagram* scraping program code is posted on *GitHub* at https://github.com/Titration/Ins-Scraping). However, data collection with the scripts was time-consuming for several reasons. First, the scripts needed to be maintained and updated frequently to cope with the platform's changes in terms of APIs or the anti-auto-data-scraping strategies. We retrieved around 80,000 *Instagram posts* for our study, but it took 5 months. While we have released our scripts as open-source, and it worked at the time of release, it too will soon require further development effort to match changes to the social media platform.

Additionally, an extensive list of available Instagram accounts and IP addresses (provided by VPNs) are also necessary to respond to blocks by the platform. Once any suspicious actions (e.g., excessive visits) are detected and identified as an auto-scraping bot, the IP address and account can be banned, temporarily or permanently, from making further requests. According to our experience, on average, we changed IP addresses three times per day and switched accounts two times per day to download 1,000 posts. Although IP addresses and social media accounts can be changed or replaced, the data delay and gaps caused by successive blocks can impact research results to different degrees (Freelon, 2018). It is also alarming for academics, particularly those early in their careers, to worry about being perceived as operating outside of a platform's legal terms and conditions.

## A better solution?

The available solutions for academics to access social media data under current restrictions are making the research field highly uneven and heterogenous (Bruns, 2019; Acker and Kreisberg, 2020). The amount of social media research *is* increasing, and it is easy to have an illusion that we are getting more data than ever before. But while the potential supply is growing—users are creating more data every day—we have access to a smaller proportion of the corpora or must rely on data collected pre-API closure, concerning researchers around issues like data representativeness, currency, and generalization of results (King and Persily, 2020). Researchers using social media appear unwilling to articulate the details of their data collection process (Weller and Kinder-Kurlanda, 2015; Poletti and Gray, 2019), either because of cumbersomeness (given the patchwork of tools available) or legal and ethical concerns. The lack of detail in method discussion is increasingly pervasive due to some new considerations: (i) published methods may not be useful for long given platform policy changes; (ii) the details may be too technical for social science audiences to follow; and (iii) there is a motivation to protect data and skills to maintain researcher competitiveness (Kinder-Kurlanda and Weller, 2014; Weller and Kinder-Kurlanda, 2015).

While the practices of data philanthropy organizations and repositories are still developing and the cost of third-party data companies are high enough to scare many researchers away (even if they *can* be enticed into collaboration), scraping tools and scripts may be the most feasible option but remain an imperfect one—the legal status of scraping is inherently problematic in addition to the privacy concerns (Bruns, 2019). Freelon (2018) noted that “researchers should bear in mind the potential (if unlikely) consequences of even small-scale terms of service violations (p. 667).” Scraping also introduces more general challenges (Weller and Kinder-Kurlanda, 2015). First, data quality is problematic in most cases where data is collected with tools without sufficient documentation, leading to opaque processes and thus weak replicability. Second, platforms may have limits on the type or the amount of data the public can access during a given period, which may result in sample biases. Third, ephemerality is a perennial challenge of social media research: platform policies can be updated at any minute, and the data can be altered or deleted (Walker, 2017). Fourth, platforms with highly restrictive APIs (e.g., *Instagram* and *Facebook*) might be avoided and more permissive APIs (e.g., *Flickr* and *Reddit*) might be preferred by researchers, causing either under- or over-representation of certain social media platforms over others, and thus certain user demographics (Barnhart, 2023).

The profound impact of social media on society suggests we should not leave addressing this problem solely to the creativity and innovation of researchers. We advocate that a better solution is a separate public research API that is not based on social media companies imposing an application-approval process. In the long run, we do not believe that **data philanthropy organizations** will be effective because they are often simply a distribution center and cannot guarantee the integrity and delivery of the data. **Social media companies** could change their one-size-fits-all approach to APIs on social media platforms to multiple ones that better serve data users with different motivations (Shtern et al., 2013; Acker and Kreisberg, 2020). However, a research API where researchers apply to the social media company for access, such as currently offered by *TikTok* is not favored, either. This grants the company complete authority to decide who can access how much data and when they can receive it. Another option is a research API gatekept by a third organization, like the Inter-university Consortium for Political and Social Research (ICPSR) at the University of Michigan Institute for Social Research (ISR) for Meta Content Library API (*Facebook* and *Instagram)*. It is unclear how different this is than the practice of data philanthropy organization, *Social Science One*. Decisions about access to such a portal should not be based on an application and review process which can favor certain research fields, regions, and researchers. However, a simple process to verify researcher status will be necessary. The platform should ideally include fact-based verification, such as verifying profile pages or email addresses of academic and research institutions, without making the topic vulnerable to rejection if it is clearly public good rather than those favored by the platforms such as commercial benefits and online democracy. As highlighted by Rieder and Hofmann (2020), it is necessary to broaden the analytical scope: data for public good should serve broader societal interests like cultural production, beyond just critical algorithm studies.

Social media companies clearly have little incentive to facilitate such a public research API (Steen-Johnsen and Enjolras, 2015). It might fulfill the company's social responsibility expectations in the social and government sphere but will not benefit (and might potentially weaken) its revenue in terms of data selling. Thus, a new governance model is required to enforce the public good values. **Government and regulators** need to intervene with laws or policies and, ideally, processes that support data sharing from social media companies to verified researchers (Vogus, 2022). An example is the Digital Services Act that was approved by the European Parliament in 2022, which provides rules to establish a mechanism for researchers to gain data access to large social media platforms and search engines (Joint Research Centre, 2023). At this stage, the effectiveness of this Act is not completely known, though *X* has taken early actions to allow EU researchers to access licensed data for DSA-related research purposes by the end of 2023 (X Developer Platform, 2024). However, the European Commission has opened formal proceedings to assess whether *X* may have breached the DSA, including concerns of suspected shortcomings in giving researchers access to X's publicly accessible data (European Commission, 2023). Nevertheless, an open gateway in Europe could facilitate transnational partnerships, allowing non-EU researchers to access data via EU collaborators.

In general, there must be clear guidelines for researchers, including how to use, store, protect, and (possibly) share data, along with the corresponding consequences for violations. Currently, such datasets sit outside of the purview of most human research ethics boards since the data is notionally available publicly. A new form of research ethics review should be developed, including setting the boundary of public data, defining fair and public-good use of social media data, and estimating the effectiveness of anonymity strategies (Taylor and Pagliari, 2018; Chen et al., 2023). A risk analysis review may also be necessary to estimate and monitor the potential harm to individual users of using social media data in specific ways and for specific purposes.

Until such a public research API can be achieved, researchers have a long and potentially dark journey ahead. There are many data collection methods at our disposal, but none of them are reliable and all come with risks such as personal legal and research quality. **Researchers** should speak frankly about the data collection process and challenges they experience, such as adding a supplementary document to disclose their detailed steps of data collection and any developed code, if applicable. Collaborative data repositories could become a feasible solution only if researchers are willing and able to share social media data with other researchers, and critically if the legal and ethical grounds can be safely and legally addressed. The precedent Sandvig v. Barr (2020) may provide an example: a district court in Columbia in the US granted researchers freedom to use data from employment websites to conduct their study. It is in the public interest to give public-good researchers legal access to high-quality social media data that is at least comparable to what commercial users have; we believe most of those contributing content to social media platforms would agree. The next thing we should do is ask them.

## Data availability statement

The Instagram scraping program code is posted on GitHub at https://github.com/Titration/Ins-Scraping. Further inquiries can be addressed to the corresponding author.

## Author contributions

YC: Conceptualization, Investigation, Methodology, Software, Writing – original draft, Writing – review & editing. KS: Funding acquisition, Supervision, Writing – review & editing. KL: Writing – review & editing. LM-P: Writing – review & editing. SX: Software, Writing – review & editing. MS: Funding acquisition, Supervision, Writing – review & editing.

## Footnotes

^1^Social Science One (2022). Available online at: https://socialscience.one/.
